# Supplementary material for: A fault-intrusion-tolerant system and deadline-aware algorithm for scheduling scientific workflow in the cloud
Source: PeerJ Comput Sci. 2021 Nov 2;7:e747. doi: 10.7717/peerj-cs.747 (PMC8576554; doi:10.7717/peerj-cs.747)
Supplement: Supplemental Information 6 [file peerj-cs-07-747-s006.zip › Myproject1.1/docs/index-files/index-19.html]

T-Index


JavaScript is disabled on your browser.


- Overview
- Package
- Class
- Use
- Tree
- Deprecated
- Index
- Help

- Prev Letter
- Next Letter

- Frames
- No Frames

- All Classes

A B C D E F G H I J L M N O P Q R S T U V W Z 


## T

Task - Class in org.workflowsim
:   Task is an extention to Cloudlet in CloudSim.

Task(int, long) - Constructor for class org.workflowsim.Task
:   Allocates a new Task object.

taskList - Variable in class org.workflowsim.ClusteringEngine
:   The task list

taskList - Variable in class org.workflowsim.WorkflowPlanner
:   The task list.

taskReceivedList - Variable in class org.workflowsim.ClusteringEngine
:   The task received list.

TaskSet - Class in org.workflowsim.clustering
:   TaskSet is a group of tasks used only in clustering

TaskSet() - Constructor for class org.workflowsim.clustering.TaskSet
:   Initialize a TaskSet object

TaskStage - Class in org.cloudbus.cloudsim.network.datacenter
:   Taskstage represents various stages a networkCloudlet can have during execution.

TaskStage(int, double, double, double, long, int, int) - Constructor for class org.cloudbus.cloudsim.network.datacenter.TaskStage


taskSubmittedList - Variable in class org.workflowsim.ClusteringEngine
:   The task submitted list.

terminateSimulation() - Static method in class org.cloudbus.cloudsim.core.CloudSim
:   This method is called if one wants to terminate the simulation.

terminateSimulation(double) - Static method in class org.cloudbus.cloudsim.core.CloudSim
:   This method is called if one wants to terminate the simulation at a given time.

TestBagofTaskApp - Class in org.cloudbus.cloudsim.examples.network.datacenter


TestBagofTaskApp(int, int, double, int, int) - Constructor for class org.cloudbus.cloudsim.examples.network.datacenter.TestBagofTaskApp


TestExample - Class in org.cloudbus.cloudsim.examples.network.datacenter


TestExample() - Constructor for class org.cloudbus.cloudsim.examples.network.datacenter.TestExample


ThrMc - Class in org.cloudbus.cloudsim.examples.power.planetlab
:   A simulation of a heterogeneous power aware data center that applies the Static Threshold (THR)
    VM allocation policy and Maximum Correlation (MC) VM selection policy.

ThrMc() - Constructor for class org.cloudbus.cloudsim.examples.power.planetlab.ThrMc


ThrMc - Class in org.cloudbus.cloudsim.examples.power.random
:   A simulation of a heterogeneous power aware data center that applies the Static Threshold (THR)
    VM allocation policy and Maximum Correlation (MC) VM selection policy.

ThrMc() - Constructor for class org.cloudbus.cloudsim.examples.power.random.ThrMc


ThrMmt - Class in org.cloudbus.cloudsim.examples.power.planetlab
:   A simulation of a heterogeneous power aware data center that applies the Static Threshold (THR)
    VM allocation policy and Minimum Migration Time (MMT) VM selection policy.

ThrMmt() - Constructor for class org.cloudbus.cloudsim.examples.power.planetlab.ThrMmt


ThrMmt - Class in org.cloudbus.cloudsim.examples.power.random
:   A simulation of a heterogeneous power aware data center that applies the Static Threshold (THR)
    VM allocation policy and Minimum Migration Time (MMT) VM selection policy.

ThrMmt() - Constructor for class org.cloudbus.cloudsim.examples.power.random.ThrMmt


ThrMu - Class in org.cloudbus.cloudsim.examples.power.planetlab
:   A simulation of a heterogeneous power aware data center that applies the Static Threshold (THR)
    VM allocation policy and Minimum Utilization (MU) VM selection policy.

ThrMu() - Constructor for class org.cloudbus.cloudsim.examples.power.planetlab.ThrMu


ThrMu - Class in org.cloudbus.cloudsim.examples.power.random
:   A simulation of a heterogeneous power aware data center that applies the Static Threshold (THR)
    VM allocation policy and Minimum Utilization (MU) VM selection policy.

ThrMu() - Constructor for class org.cloudbus.cloudsim.examples.power.random.ThrMu


ThrRs - Class in org.cloudbus.cloudsim.examples.power.planetlab
:   A simulation of a heterogeneous power aware data center that applies the Static Threshold (THR)
    VM allocation policy and Random Selection (RS) VM selection policy.

ThrRs() - Constructor for class org.cloudbus.cloudsim.examples.power.planetlab.ThrRs


ThrRs - Class in org.cloudbus.cloudsim.examples.power.random
:   A simulation of a heterogeneous power aware data center that applies the Static Threshold (THR)
    VM allocation policy and Random Selection (RS) VM selection policy.

ThrRs() - Constructor for class org.cloudbus.cloudsim.examples.power.random.ThrRs


TIME\_SHARED - Static variable in class org.cloudbus.cloudsim.DatacenterCharacteristics
:   Time-shared system using Round-Robin algorithm.

timeCommunicate - Variable in class org.cloudbus.cloudsim.network.datacenter.NetworkCloudlet


timespentInStage - Variable in class org.cloudbus.cloudsim.network.datacenter.NetworkCloudlet


timetostartStage - Variable in class org.cloudbus.cloudsim.network.datacenter.NetworkCloudlet


TopologicalGraph - Class in org.cloudbus.cloudsim.network
:   This class represents an graph containing nodes and edges, used for input with an network-layer
    Graphical-Output Restricions! EdgeColors: GraphicalProperties.getColorEdge NodeColors:
    GraphicalProperties.getColorNode

TopologicalGraph() - Constructor for class org.cloudbus.cloudsim.network.TopologicalGraph
:   just the constructor to create an empty graph-object

TopologicalLink - Class in org.cloudbus.cloudsim.network
:   This class represents an link (edge) from an graph

TopologicalLink(int, int, float, float) - Constructor for class org.cloudbus.cloudsim.network.TopologicalLink
:   creates an new link-object

TopologicalNode - Class in org.cloudbus.cloudsim.network
:   Just represents an topological network node retrieves its information from an
    topological-generated file (eg.

TopologicalNode(int) - Constructor for class org.cloudbus.cloudsim.network.TopologicalNode
:   constructs an new node

TopologicalNode(int, int, int) - Constructor for class org.cloudbus.cloudsim.network.TopologicalNode
:   constructs an new node including world-coordinates

TopologicalNode(int, String, int, int) - Constructor for class org.cloudbus.cloudsim.network.TopologicalNode
:   constructs an new node including world-coordinates and the nodeName

toString() - Method in class org.cloudbus.cloudsim.core.SimEvent


toString() - Method in class org.cloudbus.cloudsim.InfoPacket
:   Returns a human-readable information of this packet.

toString() - Method in class org.cloudbus.cloudsim.network.DelayMatrix\_Float
:   this method just creates an string-output from the internal structures...

toString() - Method in class org.cloudbus.cloudsim.network.TopologicalGraph
:   prints out all internal node and link information

toString() - Method in interface org.cloudbus.cloudsim.Packet
:   Returns a string describing this packet in detail.

toString() - Method in exception org.cloudbus.cloudsim.ParameterException
:   Returns an error message of this object.

totaldatatransfer - Static variable in class org.cloudbus.cloudsim.network.datacenter.NetworkConstants


trimZeroTail(double[]) - Static method in class org.cloudbus.cloudsim.util.MathUtil
:   Trim zero tail.

TRUE - Static variable in class org.cloudbus.cloudsim.core.CloudSimTags
:   Denotes boolean true in int value

type() - Method in class org.cloudbus.cloudsim.core.SimEvent
:   Get the user-defined tag of this event

type - Variable in class org.cloudbus.cloudsim.network.datacenter.AppCloudlet


type2record - Static variable in class org.workflowsim.failure.FailureMonitor
:   Type to a Failure Record.

TYPE\_RAW\_DATA - Static variable in class org.cloudbus.cloudsim.File
:   Denotes that the type of this file is a raw data

TYPE\_RECONSTRUCTED\_DATA - Static variable in class org.cloudbus.cloudsim.File
:   Denotes that the type of this file is a reconstructed data

TYPE\_TAG\_DATA - Static variable in class org.cloudbus.cloudsim.File
:   Denotes that the type of this file is a tag data

TYPE\_UNKOWN - Static variable in class org.cloudbus.cloudsim.File
:   Denotes that the type of this file is unknown

A B C D E F G H I J L M N O P Q R S T U V W Z

- Overview
- Package
- Class
- Use
- Tree
- Deprecated
- Index
- Help

- Prev Letter
- Next Letter

- Frames
- No Frames

- All Classes
